# Metagenomic Composition Analysis of an Ancient Sequenced Polar Bear Jawbone from Svalbard

**DOI:** 10.3390/genes9090445

**Published:** 2018-09-06

**Authors:** Diogo Pratas, Morteza Hosseini, Gonçalo Grilo, Armando J. Pinho, Raquel M. Silva, Tânia Caetano, João Carneiro, Filipe Pereira

**Affiliations:** 1 Institute of Electronics and Informatics Engineering of Aveiro, University of Aveiro, 3810-193 Aveiro, Portugal; seyedmorteza@ua.pt (M.H.); grilogoncalo31@ua.pt (G.G.); ap@ua.pt (A.J.P.); raquelsilva@ua.pt (R.M.S.); 2 Department of Electronics, Telecommunications and Informatics, University of Aveiro, 3810-193 Aveiro, Portugal; 3 Department of Medical Sciences, University of Aveiro, 3810-193 Aveiro, Portugal; 4 Institute for Biomedicine, University of Aveiro, 3810-193 Aveiro, Portugal; 5 Department of Biology, University of Aveiro, University of Aveiro, 3810-193 Aveiro, Portugal; tcaetano@ua.pt; 6 Centre for Environmental and Marine Studies, University of Aveiro, 3810-193 Aveiro, Portugal; 7 Interdisciplinary Centre of Marine and Environmental Research, University of Porto, 4450-208 Matosinhos, Portugal; joaomiguelsov@gmail.com (J.C.); fpereirapt@gmail.com (F.P.)

**Keywords:** ancient DNA, composition analysis, polar bear, metagenomics, relative compression

## Abstract

The sequencing of ancient DNA samples provides a novel way to find, characterize, and distinguish exogenous genomes of endogenous targets. After sequencing, computational composition analysis enables filtering of undesired sources in the focal organism, with the purpose of improving the quality of assemblies and subsequent data analysis. More importantly, such analysis allows extinct and extant species to be identified without requiring a specific or new sequencing run. However, the identification of exogenous organisms is a complex task, given the nature and degradation of the samples, and the evident necessity of using efficient computational tools, which rely on algorithms that are both fast and highly sensitive. In this work, we relied on a fast and highly sensitive tool, FALCON-meta, which measures similarity against whole-genome reference databases, to analyse the metagenomic composition of an ancient polar bear (*Ursus maritimus*) jawbone fossil. The fossil was collected in Svalbard, Norway, and has an estimated age of 110,000 to 130,000 years. The FASTQ samples contained 349 GB of nonamplified shotgun sequencing data. We identified and localized, relative to the FASTQ samples, the genomes with significant similarities to reference microbial genomes, including those of viruses, bacteria, and archaea, and to fungal, mitochondrial, and plastidial sequences. Among other striking features, we found significant similarities between modern-human, some bacterial and viral sequences (contamination) and the organelle sequences of wild carrot and tomato relative to the whole samples. For each exogenous candidate, we ran a damage pattern analysis, which in addition to revealing shallow levels of damage in the plant candidates, identified the source as contamination.

## 1. Introduction

Due to constant low temperatures, glacial ice and permafrost environments provide potential conditions for long-term survival of DNA molecules, increasing the likelihood of ancient DNA (aDNA) authentication [1,2,3,4]. The jawbone fossil of an ancient polar bear (*Ursus maritimus*) is one of the best-preserved fossils discovered so far given its age [5]. This fossil was collected in the Poolepynten region of Svalbard, Norway, and estimated to be 110,000 to 130,000 years old. The sequencing of the ancient polar bear (PB) genome [6,7] and its comparison against the genomes of other bears revealed an evolutionary history characterized by gene flow across species [8], allowed the identification of an endogenous bear retrovirus [9], and provided evidence of past climate change [7].

The sequencing of ancient species allows DNA of endogenous and exogenous origins to be identified. When target enrichment sequencing is not applied [4], the machine generates a large volume of exogenous DNA, most of which is microorganic [10]. In addition to rare examples of addition of exogenous ancient microorganisms [11,12], contamination is known to be a primary cause of inclusion of exogenous microorganisms [1,13].

Employing affordable computational resources to efficiently split the exogenous from endogenous DNA, including the classification of exogenous content, is a complex challenge. Filtering undesired sources from the primary target enables improvement of the quality of assemblies and, thus, data analysis. More importantly, such filtering allows extinct and extant species to be detected without the need to resort to a specific or new sequencing run. The identification of exogenous organisms is not trivial given the nature and degradation of the samples and the evident need to use efficient computational tools. There are many computational tools for metagenomic composition analysis [14,15,16,17,18,19,20,21,22,23,24,25,26,27,28,29,30,31,32,33,34,35], based on both alignment and alignment-free techniques [36,37]. For a relevant comparison, see [38].

In aDNA metagenomics, every algorithm aims to be both highly sensitive and fast. However, algorithms that are highly sensitive are usually slower and introduce a new kind of concern: overestimation of similarity [39]. High sensitivity occurs when classifiers have high capabilities and diversity, for example, when they are capable of dealing with genomic rearrangements (inversions, translocations, duplications, fusions, and fissions), high stochastic variation (especially high levels of substitution), high heterogeneity (high alternation between high- and low-complexity regions), and short fragments (reads) displayed in an arbitrary order [40]. For this purpose, a simple high-order k-mer model is generally not enough to accomplish high sensitivity. When using multiple models of different depths, the question becomes how to decide which model better represents a particular region. Should we also measure the information needed to describe the model selection? Recently, we answered these questions [39] using the Normalized Relative Compression (NRC) [41,42]. In fact, we showed that, if the models are not qualified to handle a specific region, then the information required to measure similarity is transferred to the selection of the used model. In other words, if we ignore side information in multiple stochastic models and choose only the correct model, then there is a high probability that the prediction will remain accurate, while the decision becomes highly complex. This reflects the high importance of working with measures that do not overestimate similarity while using multiple predictors in the search to increase sensitivity.

We created a highly sensitive tool, FALCON-meta [39], where the number of models (predictors) and parameters sets the precision required, in balance with the available RAM. Moreover, the tool exhibits competitive speed relative to most of the existing tools. FALCON-meta is a tool that can, efficiently, operate in general metagenomics studies. However, given the ability to increase the model’s sensitivity to values that, as far as we know, have not been attained by any other method without similarity overestimation, it is the natural candidate for efficient application in ancient metagenomic studies, especially when the reads have very short lengths, duplications, and inversions.

In this paper, we study the metagenomic composition of a sequenced polar bear tooth sample, using the FALCON-meta tool. A preliminary metagenomic analysis of 454 sequence reads was previously reported, although it only included the sequencing of the mitogenome [6]. Here, we consider the whole-genome. We follow the underlying protocols of validation of high throughput sequencing and microbial forensics applications [43], with custom additions for the supported framework. First, we present the pipeline used, along with the preparation of the sample, database creation, and models and parameters chosen to run FALCON-meta. Then, we run the analysis and split the results into the mitochondrial, plastidial, archaeal, bacterial and viral genomes. We identify several potential inclusions of organisms in the samples. We classify each candidate according to ancient or present contamination, supported by a consistent damage pattern analysis [44,45].

## 2. Methods

We downloaded the genome sequence data of an ancient Poolepynten *Ursus maritimus* (PUM) sample using the following accession codes: SRR518649, SRR518651, SRR518654, SRR518656, SRR518657, SRR518659, SRR518704, SRR518705 and SRR518706. Given the different dates relative to the majority of runs, we chose not to include the SRR518655 and SRR525046 runs. The PUM sample contains 349 GB of WGS nonamplified shotgun data, which includes a 110,000- to 130,000-year-old polar bear specimen from Svalbard, Norway. The sample was sequenced with an Illumina HiSeq 2000 [7]. The PUM sample contains 1,342,773,480 paired-end (PE) reads with 101 base pairs and a quality-score range of 39.

**Figure 1:**
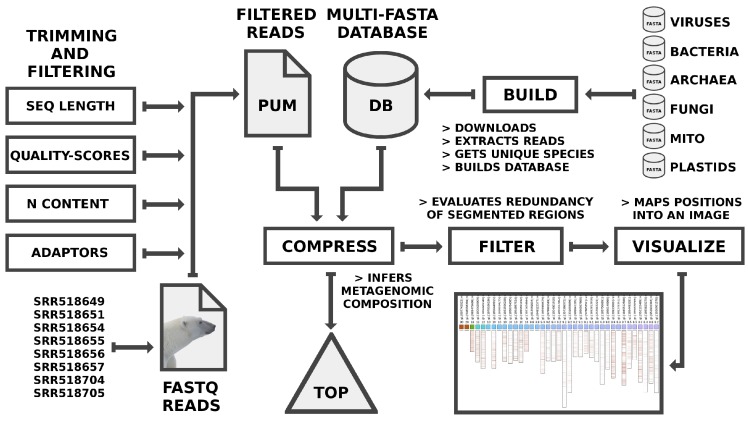
Pipeline for the analysis of metagenomic composition using the ancient sample (PUM) and a database containing several reference organisms as input, where MITO stands for mitochondrial genomes. The BUILD phase was conducted according to Section 2.2. The COMPRESS phase is conducted using the computation of FALCON-meta. The FILTER phase was a control to detect self-redundancy and distribution.

Figure 1 shows the pipeline used in the analysis. The FASTQ reads were trimmed and filtered using AdapterRemoval v2 [46] (see Section 2.1). The database was built using the reference genomes from viruses, bacteria, archaea, fungi, mitochondria and plastids (see Section 2.2). FALCON-meta performs the compression, filtering, and visualization operations (see Section 2.3).

Note that all results presented in this paper can be fully replicated, with a Linux machine using the scripts provided in the repository https://github.com/pratas/bear. These scripts include the automatic installation of the tools, download of the files, computation, and visualization of the results. For further damage pattern analysis, we used BWA [25], Bowtie [24], SAMtools [47] and mapDamage2 [45], according to [48,49]. The Bioconda tool [50] was used to install BWA, Bowtie, SAMtools and mapDamage2.

### 2.1. Filtering and Trimming Reads

Filtering and trimming reads are essential to guarantee high quality and accurate analysis. The PUM reads were filtered and trimmed using AdapterRemoval v2 [46]. For each file pair, we ran AdapterRemoval, which trimmed N symbols, removed entries with qualities below a particular score, and excluded reads with a DNA sequence size of less than 25 bases. Then, we merged the files into the PUM.fq file. With this procedure, we have discarded 43% of the reads. The final PUM file contained 773,794,456 reads and had a total size of approximately 160 GB.

### 2.2. Building the Database

To build the database (DB), we downloaded several domains/kingdoms/types of data sets from the NCBI database (27 April 2018) using specific scripts. Table 1 includes the datasets, their characteristics, and the names of the scripts for download.

Then, we removed the *hypothetical* and partial sequences; specifically, we selected only complete genomes for the final database.

### 2.3. Running FALCON-Meta

We used the following parameters to run FALCON-meta: -n 8 -l 45 -t 500 -F -Z -c 250. This mode includes the automatic parameterization of a relative compressor [51] that applies soft-blending [52], with a decaying forgetting factor [53], between four context models (CMs) [52,54] and one tolerant CM [55]. The decaying factor used was 0.95, and the cache hash was 250 [56]. The models have the following parameters:

1. **Tolerant CM**: depth: 20, alpha: 0.1, tolerance: 5;
2. **CM**: depth: 20, alpha: 0.005, inverted repeats: yes;
3. **CM**: depth: 14, alpha: 0.01, inverted repeats: yes;
4. **CM**: depth: 11, alpha: 0.1, inverted repeats: no; and
5. **CM**: depth: 6, alpha: 1, inverted repeats: no.

The cooperation between these models acts as a very powerful data mining system. For detailed information on the parameters and their meanings, see [39,51].

Generally, the FALCON-meta tool uses the designated models to learn the internal features of the data from the total FASTQ reads. Then, the tool freezes the accumulated knowledge, allowing the system to exclusively estimate further probabilities using read-only access. Finally, it estimates the amount of new information seen when compressing each reference sequence independently. For each measure, the length of the sequence (in the appropriate logarithmic scale) is used to normalize the value. The resulting value represents the NRC, which given the respective complement, with renormalization, provides the Normalized Relative Similarity (NRS). The NRS is an estimate of how similar (exclusively) a string is to another, according to the respective scale. For an extensive formal definition (see [39,57]).

The FALCON-meta package, as shown in Figure 1, includes programs to map (compress), filter (enabling localization of similar regions), and visualize the results. The commands used for all the package programs were the following:

./FALCON -v -n 1 -t 800 -l 45 -F -Z -c 250 -y complexity.com PUM.fq DB.fa
./FALCON-FILTER -v -F -sl 0.001 -du 20000000 -t 0.5 -o positions.csv complexity.com
./FALCON-EYE -v -e 500 -s 4 -o top.svg positions.csv

For visualization enhancement purposes, we have split the content of the images according to the different domains and natures of the databases.

## 3. Results

All computations were run on an Ubuntu Linux computer with a 2.13 GHz core and a maximum RAM of 34.3 GB. Utilizing this machine, without parallelization, the computation of the metagenomic composition analysis of the PUM dataset required almost 1953 min (32.55 h).

To permit easier visualization, we have split the analysis of the PUM sample based on the characteristics of the reference sequences, specifically by dividing the images into mitochondrial, plastidial, archaeal, bacterial, and viral types.

**Figure 2:**
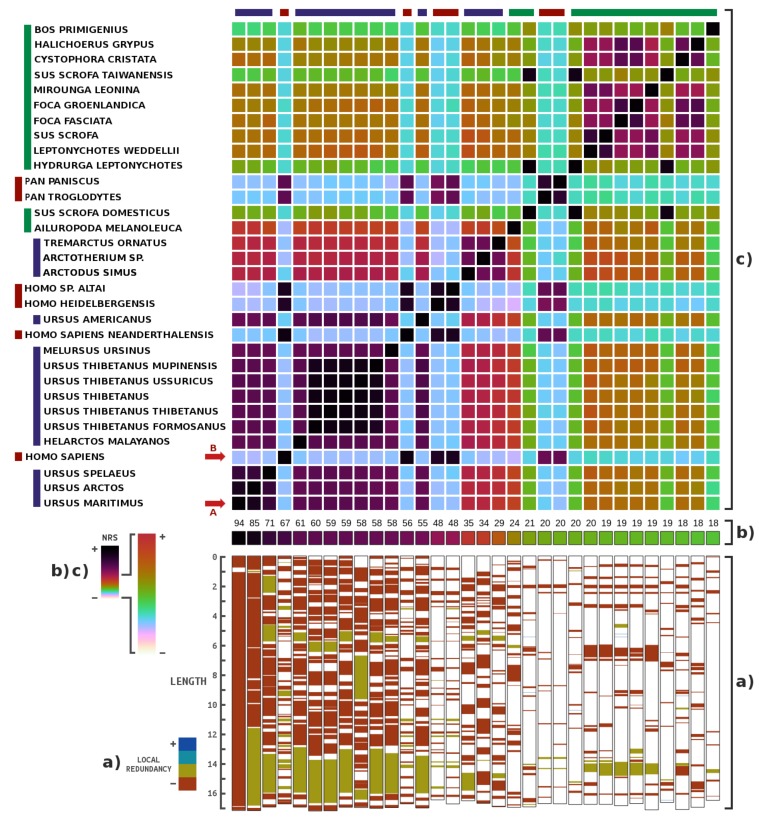
Metagenomic composition analysis of the PUM (Poolepynten *Ursus maritimus*) sample, specifically for mitochondrial genomes: (**b**) the percentage of the highest NRS entries in descending order (from left to right), according to the order of the names on the left (from bottom to top); (**a**) locations where the mitochondrial genomes are similar relative to the reads, as well as their respective redundancies, mapped with four colours; and (**c**) intra-similarities of the mitochondrial genomes based on the NRS. The matrix appears to be symmetric because the sizes of the samples are approximately the same. Length is presented in 10^3^. Letters (in red) identify potential genomes contained in the samples.

Figure 2 depicts the results of the highest NRS values for mitochondrial genomes. As expected, *Ursus maritimus* has the highest similarity, since the PUM sample contains an ancient version of the bear. Several bear genomes appear to have high NRS values, although these NRS values are below that of *U. maritimus*, namely, *U. arctos*, *U. spelaeus*, *H. malayanos*, *U. thibetanus*, *M. ursinus* and *U. americanus*. Based on the database intra-similarity (Figure 2c), these are naturally very similar mitochondrial genomes; therefore, given the high values, we can discard their presence in the samples. In fact, this analysis is comparable to a phylogenomic analysis [58].

Additionally, outside the bear lineage, there is an unusually high NRS value for *Homo sapiens*. Other high NRS values within *Homo* are also present, although based on the database intra-similarity, they appear to be similar to the modern human. This result seems to be a case of human contamination. In fact, human contamination was previously reported in an ancient mitochondrial sample of *U. maritimus* [6]. In this sample, the same occurs but at the whole genome level.

Regarding the NRS values of the other organisms, for example, pig, seal, and cow, as shown in Figure 2c, there is a high degree of intra-similarity with top organisms such as the modern human and the bear. To understand the impact of these similarities on the analysis, we filtered the PUM reads with a similarity relative to the *Ursus maritimus* reference sequence of over 0.95. Since we wanted to guarantee the presence of flanking regions and possible evolutionary regions, we accepted a 0.05 read similarity.

**Figure 3:**
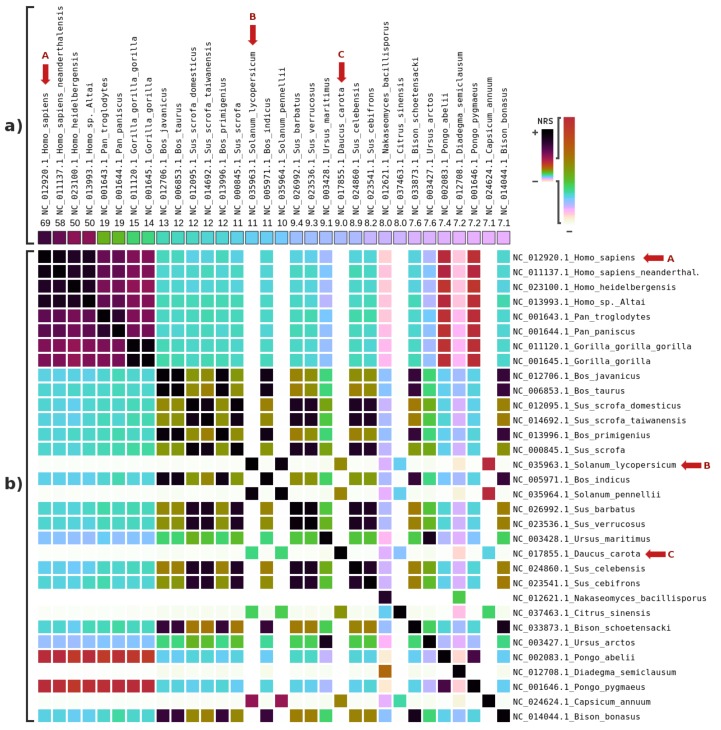
Metagenomic (mitochondrial) composition analysis of the reads, from the PUM sample, that did not exceed a similarity threshold relative to the *Ursus maritimus* reference sequence of 0.95: (**a**) the percentage of the highest NRS entries in descending order (from left to right), according to the order of the names at the top; and (**b**) the intra-similarities of the mitochondrial genomes based on the NRS. Letters (in red) identify potential genomes contained in the samples.

Accordingly, Figure 3 depicts the results of the highest NRS values for mitochondrial genomes relative to the filtered samples. The results show the presence of the modern human and respective similar genomes, increasing the likelihood that contamination occurred. Several plant mitogenomes (marked with B and C) from *Solanum lycopersicum* (tomato) and *Daucus carota* (wild carrot) were also present, with similarities among them.

**Figure 4:**
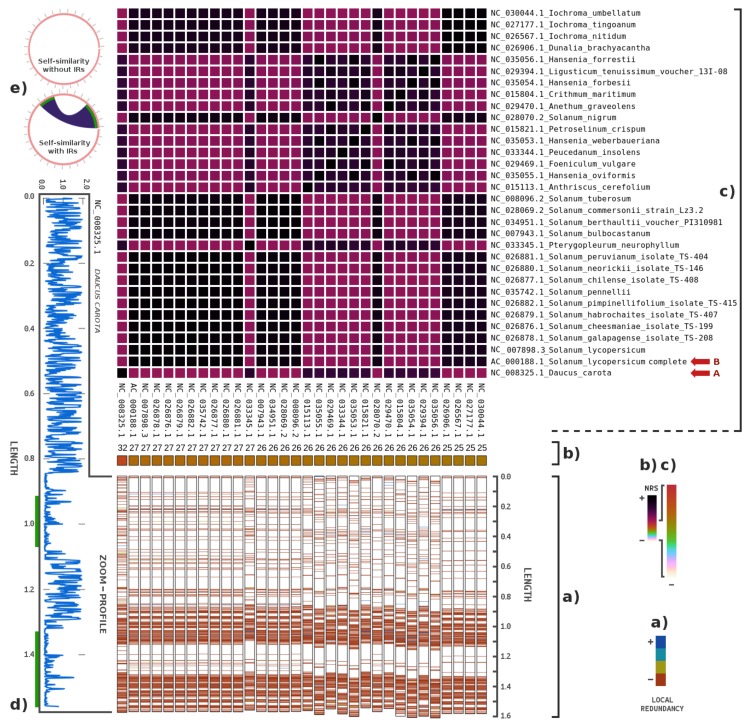
Metagenomic composition analysis of the PUM sample, particularly for plastid genomes: (**b**) The percentage of the highest NRS entries in descending order (from left to right), according to the order of the names on the right (from bottom to top). (**a**) Locations where the plastid genomes are similar relative to the reads, as well as their respective redundancies, mapped with four colours. (**c**) The intra-similarities of the plastid genomes based on the NRS. The matrix appears to be symmetric because the sizes of the samples are approximately the same. Length is presented in 10^5^. The sequence identifiers represent the names of the plastids. (**e**) The circular maps indicate where similarities are located among parts of the *Daucus carota* sequence. (**d**) The complexity profile of the sequence relative to that of the PUM sample [59]. Letters (in red) identify potential genomes contained in the samples.

Figure 4 depicts the results of the highest NRS values for plastid genomes. The sequence with the highest NRS (≈32%) stands for the chloroplast of *D. carota* (marked with A), also known as wild carrot. In fact, the mitochondrial sequence of a *D. carota* subspecies (NC_017855.1) was previously used in a mitochondrial metagenomic analysis, and an NRS of 9% was obtained. Additionally, the chloroplast of *S. lycopersicum* (marked with B) had an NRS near 27%. The database intra-similarity values revealed high similarity between these plastid sequences.

Given the NRS of *D. carota* and the relative difference of this species from others, we focused on this species. To remove the *noise* from the samples, we filtered only the reads with similarity to several carrot chloroplast sequences (using script runCarrots.sh for replication). Then, we ran FALCON-meta, but instead of the whole PUM sequence, we used only the filtered reads. We found an NRS of 63% in the complete *D. carota* chloroplast genome (155,911 bases). Since the NRS is an approximation of the similarity value without overestimation, the real value may be higher.

The *D. carota* relative complexity profile, as shown in Figure 4d, has high representability given the PUM sample. There are two sub-regions in the plastid sequence that are similar due to inverted repeats (Figure 4e). For a study on inverted repeats in multiple genomes, see [60]. Except for the inversion, the plastid of *D. carota* is generally highly complex, which means that the inclusion of this sequence in the sample, although unlikely, may be due to its similarity to another organism or to it being contained in the sample. However, *S. lycopersicum* was also contained in the sample, although in this case, the sample was from the mitochondria (Figure 3a).

**Figure 5:**
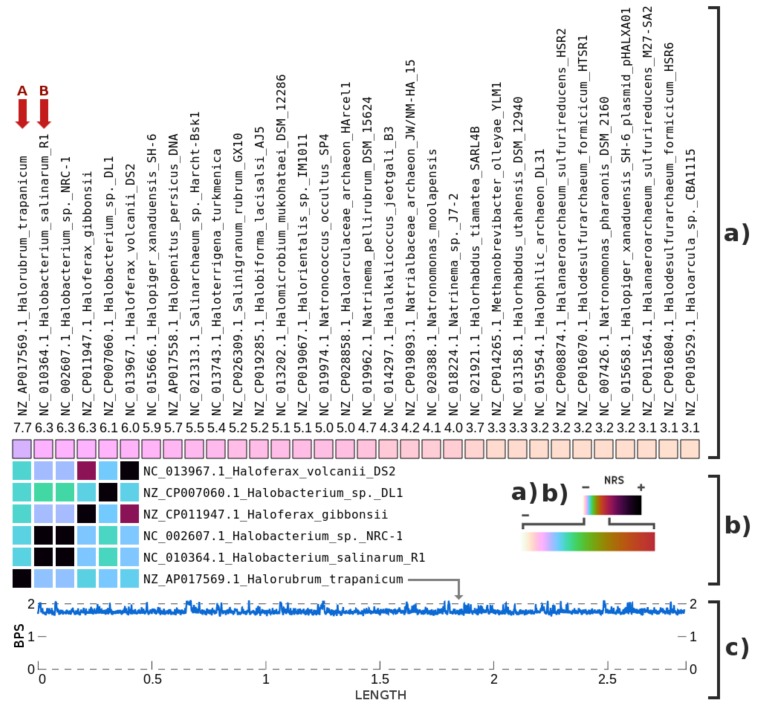
Metagenomic composition analysis of the PUM sample, specifically for archaeal genomes: (**a**) The percentage of the highest NRS entries in descending order (from left to right), according to the order of the names at the top (from bottom to top). (**b**) The Top 6 archaeal genome intra-similarities based on the NRS. The names of the archaeal genomes and their respective sequence identifiers are both represented. Letters (in red) identify potential genomes contained in the samples. (**c**) The complexity profile of *Halorubrum trapanicum* relative to the sample.

The highest NRS values for archaeal genomes (Figure 5) matched members of halophilic Archaea that typically live in saline environments. *Halorubrum trapanicum* (denoted with A) and *Halobacterium salinarum* shared some similarity. Hence, we discarded *H. salinarum* from the sample. In Figure 5c, we present a complexity profile that shows the *H. trapanicum* similarity (the complement of complexity) relative to the PUM reads. Although the pattern seems uniformly distributed, the low similarity does not permit the inference of any consistent source. The archaeon my be unknown, somewhat mutated or similar to another organism in the database.

**Figure 6:**
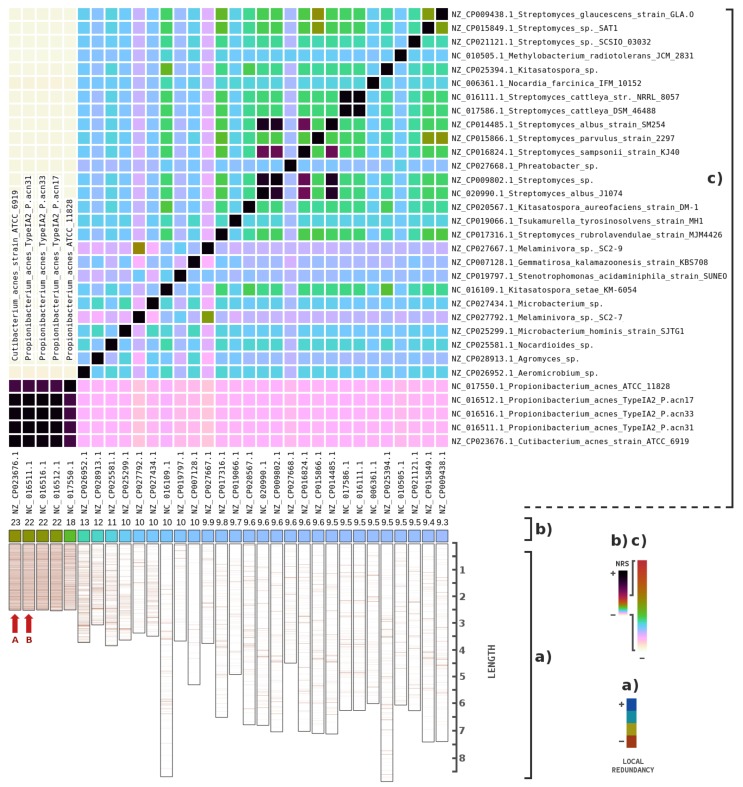
Metagenomic composition analysis of the PUM sample, specifically for bacterial genomes: (**a**) the local similarity (redundancy) of the highest NRS entries in descending order (from left to right), according to the order of the names at the top (from bottom to top). The scale is in megabases; (**b**) the percentage of the highest NRS entries in descending order (from left to right); and (**c**) the bacterial genome intra-similarities based on the NRS. The names of the bacteria and the sequence identifiers are both represented. Letters (in red) identify potential genomes contained in the samples.

Figure 6 depicts the highest NRS values for bacterial genomes. There was high similarity between the references of *Cutibacterium acnes* and *Propionibacterium acnes*. Although these bacteria have different names, they were recently taxonomically classified as the same species [61]. Generally, these bacteria are detected in ancient and post-mortem samples. Additionally, the similarity map (Figure 6a) shows a uniform distribution pattern, reinforcing the potentiality for these references to be contained within the PUM sample, with a high probability of being contaminants.

**Figure 7:**
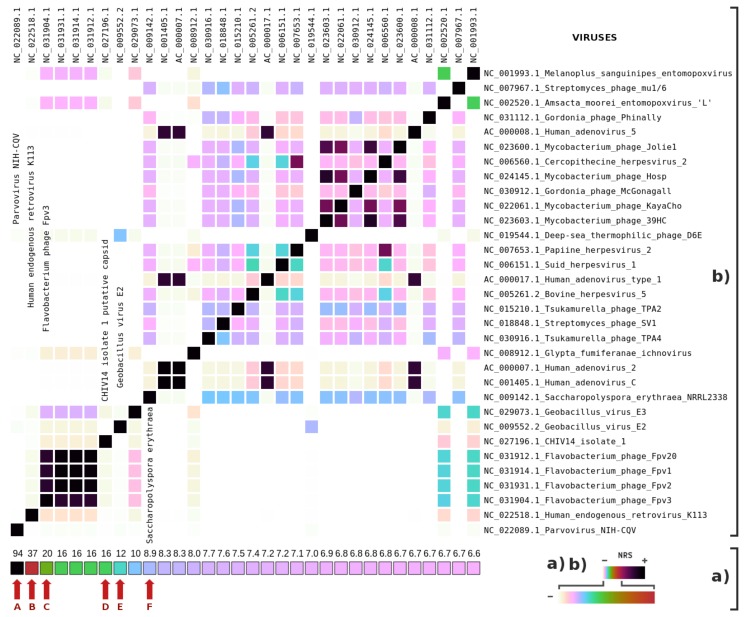
Metagenomic composition analysis of the PUM sample, specifically for viral genomes: (**a**) the percentage of the highest NRS entries in descending order (from left to right), according to the order of the names at the top; abd (**b**) the viral genome intra-similarities are based on the NRS. The names of the viruses and the sequence identifiers are both represented. Letters (in red) identify potential genomes contained in the samples.

Figure 7 depicts the highest NRS values for viral genomes. There was a very high degree of similarity with the reference of *Parvovirus* NIH-CQV (marked with the letter A). We filtered the reads corresponding to the virus and then assembled it using SPAdes [62]. Finally, we used BLASTn [63] to align the assembled sequence with the reference of *Parvovirus* NIH-CQV (NCBI). We found 99% similarity (99% identity) in the aligned genome. This analysis gives strong evidence that the virus is included in the PUM sample due to contamination. In fact, *Parvovirus* NIH-CQV has been widely associated with laboratory contamination [64,65].

The results also revealed a high similarity to the human endogenous retrovirus K113 (marked with the letter B). This finding is not surprising since we know from Figure 2 that the sample has modern human DNA contamination. Remarkably, a BLASTn [63] analysis showed 70% similarity and 4% identity between the human sequence and a bear retroviral sequence, using a search conducted with only data from the NCBI.

In contrast, we found a 20% similarity to the flavobacterium phage Fpv3 [66] (marked with tge letter C), a phage with 88,421 bases. Several distinct organisms had some degree of similarity without having significant similarity to the other organisms, namely, *Geobacillus* and *Saccharopolyspora erythraea*, which makes them potential targets for future analysis.

Given all the candidates, the problem now becomes how to classify organismal DNA as ancient or arising from contamination. Fortunately, for this specific case, ancient DNA reveals damage patterns [67,68]. These patterns have characteristics that distinguish ancient from contaminant species, such as a specific increase in substitutional alterations in the tips of the reads (relative to a modern reference) [69]. The most effective programs to split or classify ancient DNA from contaminants are PMDtools [44] and mapDamage [45].

As a control, we ran a pattern analysis of the ancient bear genome. Naturally, it revealed ancient characteristics, given the high levels of C-T and G-A substitutions in the tips of the reads (Supplementary Figures S1–S9). Unlike the bear, and excluding the plants, all the candidates exhibited an absence of damage (Supplementary Figures S1–S9). These properties are consistent with contamination. Regarding the plants, there were shallow (very low) levels of damage with high noise, which prevented further conclusions.

## 4. Discussions

Consider the analogy where a polar bear genome, made of small pieces of iron, is distributed randomly inside a haystack. The haystack is full of straw. How can we find and assemble all the parts of the iron polar bear? Currently, to address this subject, most paleogenomics researchers opt to use a single magnet. Although the magnet attracts the majority of the pieces, some are dropped, for example, because the magnet’s volume is small, the magnet contains a different geometry, or there are external fields.

To overcome this limitation, we need to enhance the identification process, specifically by increasing its sensitivity. Sensitivity is related to the development of models with the capability for more accuracy. For this purpose, we use multiple magnets of different sizes (k-mers) and geometry (types, such as tolerant models or *regular* models and the use or absence of inverted repeats).

If a model uses a lower k-mer, does it not become more sensitive? In some way, it does, but it will introduce more noise. Generally, a short k-mer model has a lower modelling capability than a higher one (for example, a shorter memory or shorter precision), while, although a higher k-mer model can discriminate the data better than a shorter one, it is not able to work with small characteristics. Therefore, the competition or cooperation between different models better suits the analysis.

Now, the question becomes how to select the model that best represents a specific region. If we give the same weight to each magnet, then they will try to attract the majority of the pieces. The competition approach will consume the energy of a substantial part of the system. This consumption happens because, if the magnets are in opposing places, they will disrupt the attraction of others, roughly sustaining the pieces of iron in the same area or increasing the attraction time. This process represents the analogy of overestimation. Here, we need to define which magnet is more suitable for attracting specific pieces. FALCON-meta uses all the magnets, although the magnets assume different degrees of importance. The magnets are supervised by an automatic mechanism that attributes importance according to the performance of the latest attraction records. Notice that the use of multiple models without equilibrium is related to higher uncertainty.

Consider now that the haystack has other types of objects with ferrite, representing other organisms (contamination). In this case, we will probably attract other genomes. If the genomes have common properties, then we will attribute the source of specific parts of the polar bear, especially when we have multiple copies (coverage) with high stochastic variation. There are two main ways of addressing this problem, namely, by competitive matching [17,70] and by database intra-similarity analysis [39]. We prefer to deal with the database intra-similarity analysis because sometimes samples (parts of the genomes) do not represent the whole genomes. For example, although a horizontal gene can be part of a sample and, in a competitive approach, mapped onto a specific genome, the whole genome may be different, especially when we are dealing with very similar organisms. Other known problems are segmental duplications and inversions [71,72].

In this work, we have also shown the importance of addressing the metagenomic analysis before the analysis of the target genome. For example, what is the consistency of an investigation of an ancient hominin when there is human contamination in the samples? Here, even enrichment and damage pattern analysis cannot solve the problem entirely. Although there have been many advances in quality control [13], the challenge remains unsolved and is, perhaps in some parts, undecidable. Therefore, the awareness and discrimination of contaminant organisms in samples are proof of analysis integrity and quality.

Using the PUM sample, we identified multiple organisms of different domains and kingdoms with a high probability of being contained in the sample. We analysed the damages patterns of the reads relative to each respective candidate reference. Neglecting the shallow (very low) levels of damage in the plants, the absence of damage permitted us to classify the candidates as contaminants.

The high similarity of the mitochondria and chloroplasts of the plants produced the results that need further exploration, namely, there is a higher similarity of the reads to the tomato mitochondria than to the wild carrot and a higher similarity of the reads to the wild carrot plastid than to the tomato. We may have identified insights into an unreported genome plant with properties similar to those of the identified plants. Although not reported here, we also found a high similarity of the whole genome of a maize reference to the reads. We are not able to proceed to a final analysis based on damage pattern because the references may not be the most representative.

The human and *Parvovirus* contaminations are some of the examples with the highest probability. Although the *Parvovirus* is small and can be removed from the samples, the human retrovirus is more challenging since it may have some short regions similar to bear retrovirus. Usually, these organisms are undesirable for target analysis purposes. The awareness of their presence in the samples provides a way to filter them, improving the quality of the analysis. Note that the mentioned application is not limited to ancient DNA but can also be used in studies with broader analysis since it allows the filtering of exogenous sources that may have been incorporated into reference assemblies from whole genome sequence data.

The abundance of publicly available digital samples is a considerable repository of novel and variant genomic systems collected from distinct environments. These organisms accompany target sequenced organisms without having this purpose and are considered contamination. However, contaminant organisms can also be a source of inexpensive sequencing, which is conditioned on efficient and fast computational methods to reveal them.

## 5. Conclusions

The number of studies on aDNA is steadily increasing, supporting the capability of combining aDNA with archaeological findings to increase knowledge of our ancestors’ history. The Poolepynten polar bear jawbone is one of the best-preserved fossils from the Pleistocene. With the publicly available Poolepynten polar bear sequence reads, we sought to analyse metagenomic composition.

FALCON-meta was used to infer metagenomic composition automatically. We used the intra-similarity of the database to find the organisms most likely to be contained in the samples. We identified multiple potential genomes, showing that these samples contain significant amounts of exogenous genomes of different species. Some examples are genomes from a modern human (and the respective endogenous retrovirus), *Parvovirus*, *Cutibacterium*, *Geobacillus*, *Flavobacterium*, and a plant similar to *D. carota* (wild carrot) and *S. lycopersicum* (tomato). Curiously, the results revealed a lower similarity to the tomato plastid sequence than to that of the wild carrot and a higher similarity to the tomato mitochondrial sequence than to that of the wild carrot, leading us to think that we sampled a plant with an unsequenced genome.

For each potential organism, we ran a damage pattern analysis, identifying the genomes as sources of contamination based on their absence of deamination characteristics. The only exceptions were the plant organelles, which exhibited shallow levels of damage. Generally, the ancient polar bear sample contained multiple sequences from other sources classified as present-day contaminants. An awareness of these sequences in the sample provides a way to attain integrity and improve the quality and consistency of the analysis.

## Figures and Tables

**Table 1:** Characteristics of the database before filtering and the corresponding downloaded scripts.

Domain/Kingdom/Type	Number of Sequences	Length	Script
Viruses	9626	338 MB	DownloadViruses.pl
Archaea	40,322	3.4 GB	DownloadArchaea.pl
Bacteria	2,245,000	130 GB	DownloadBacteria.pl
Fungi	2,205,000	11 GB	DownloadFungi.pl
Mitochondrion v2	8670	212 MB	DownloadMTV2.sh
Plastid v2	2938	308 MB	DownloadPlastidV2.sh
Total (DB)	4,511,556	145.2 GB	

## Supplementary Materials

The following are available online at http://www.mdpi.com/2073-4425/9/9/445/s1. Figure S1. Damage patterns for the reference *U. maritimus* computed with mapDamage. The four upper mini-plots (left panel) show the base frequency outside and in the read (the open grey box corresponds to the read). The bottom plots are the positions’ specific substitutions from the 5″ (left) and the 3″ end (right). The empirical misincorporation frequencies and simulated posterior predictive intervals from the fitted model are depicted at the right panel. For more information, see https://ginolhac.github.io/mapDamage/. Figure S2. Damage patterns for the reference *H. sapiens* computed with mapDamage. The four upper mini-plots (left panel) show the base frequency outside and in the read (the open grey box corresponds to the read). The bottom plots are the positions’ specific substitutions from the 5″ (left) and the 3″ end (right). The empirical misincorporation frequencies and simulated posterior predictive intervals from the fitted model are depicted at the right panel. For more information, see https://ginolhac.github.io/mapDamage/. Figure S3. Damage patterns for the reference *C. acnes* computed with mapDamage. The four upper mini-plots (left panel) show the base frequency outside and in the read (the open grey box corresponds to the read). The bottom plots are the positions’ specific substitutions from the 5″ (left) and the 3″ end (right). The empirical misincorporation frequencies and simulated posterior predictive intervals from the fitted model are depicted at the right panel. For more information, see https://ginolhac.github.io/mapDamage/. Figure S4. Damage patterns for the reference *H. trpanicum* computed with mapDamage. The four upper mini-plots (left panel) show the base frequency outside and in the read (the open grey box corresponds to the read). The bottom plots are the positions’ specific substitutions from the 5″ (left) and the 3″ end (right). The empirical misincorporation frequencies and simulated posterior predictive intervals from the fitted model are depicted at the right panel. For more information, see https://ginolhac.github.io/mapDamage/. Figure S5. Damage patterns for the reference *D. carota* computed with mapDamage. The four upper mini-plots (left panel) show the base frequency outside and in the read (the open grey box corresponds to the read). The bottom plots are the positions’ specific substitutions from the 5″ (left) and the 3″ end (right). The empirical misincorporation frequencies and simulated posterior predictive intervals from the fitted model are depicted at the right panel. For more information, see https://ginolhac.github.io/mapDamage/. Figure S6. Damage patterns for the reference *S. lycopersicum* computed with mapDamage. The four upper mini-plots (left panel) show the base frequency outside and in the read (the open grey box corresponds to the read). The bottom plots are the positions’ specific substitutions from the 5″ (left) and the 3″ end (right). The empirical misincorporation frequencies and simulated posterior predictive intervals from the fitted model are depicted at the right panel. For more information, see https://ginolhac.github.io/mapDamage/. Figure S7. Damage patterns for the reference *CHIV14* computed with mapDamage. The four upper mini-plots (left panel) show the base frequency outside and in the read (the open grey box corresponds to the read). The bottom plots are the positions’ specific substitutions from the 5″ (left) and the 3″ end (right). The empirical misincorporation frequencies and simulated posterior predictive intervals from the fitted model are depicted at the right panel. For more information, see https://ginolhac.github.io/mapDamage/. Figure S8. Damage patterns for the reference *Flavobacterium* phage computed with mapDamage. The four upper mini-plots (left panel) show the base frequency outside and in the read (the open grey box corresponds to the read). The bottom plots are the positions’ specific substitutions from the 5″ (left) and the 3″ end (right). The empirical misincorporation frequencies and simulated posterior predictive intervals from the fitted model are depicted at the right panel. For more information, see https://ginolhac.github.io/mapDamage/. Figure S9. Damage patterns for the reference *Geobacillus* computed with mapDamage. The four upper mini-plots (left panel) show the base frequency outside and in the read (the open grey box corresponds to the read). The bottom plots are the positions’ specific substitutions from the 5″ (left) and the 3″ end (right). The empirical misincorporation frequencies and simulated posterior predictive intervals from the fitted model are depicted at the right panel. For more information, see https://ginolhac.github.io/mapDamage/.

## Abbreviations

The following abbreviations are used in this manuscript:

aDNA	ancient DNA
CM	Context Model
DB	Database
DNA	Deoxyribonucleic acid
NRC	Normalized Relative Compression
NRS	Normalized Relative Similarity
PB	Polar Bear
PE	Paired Ends
PUM	*Poolepynten Ursus maritimus* (ancient Polar Bear)
RAM	Random Access Memory
