# Supplementary material for: Metagenomic Composition Analysis of an Ancient Sequenced Polar Bear Jawbone from Svalbard
Source: Genes (Basel). 2018 Sep 6;9(9):445. doi: 10.3390/genes9090445 (PMC6162538; doi:10.3390/genes9090445)
Supplement: Supplementary file 1 [file genes-09-00445-s001.pdf]

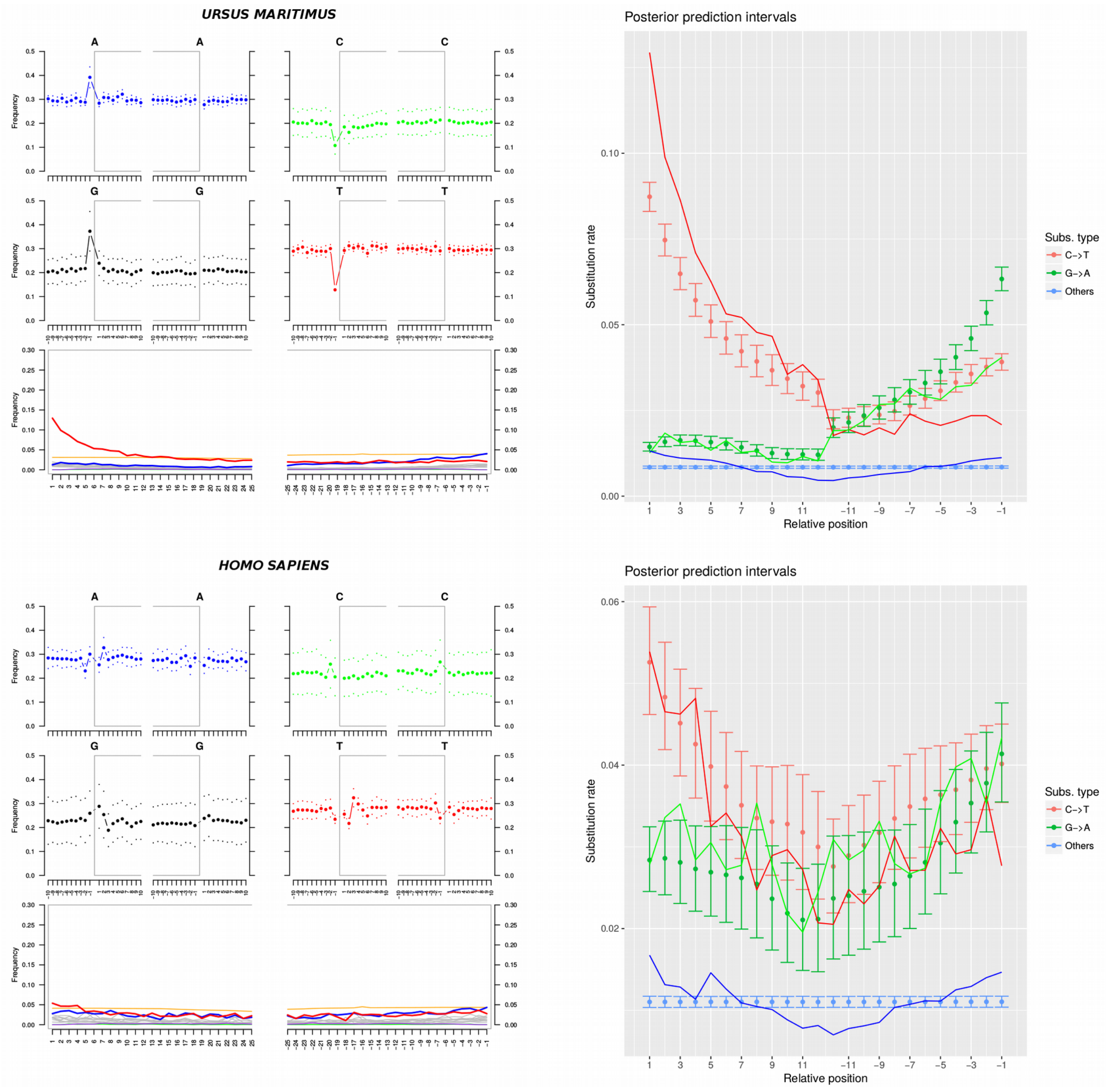
URSUS MARITIMUS
A
A
C
C
G
G
T
T
Frequency
Posterior prediction intervals
Substitution rate
Relative position
Subs. type
C->T
G->A
Others
HOMO SAPIENS
A
A
C
C
G
G
T
T
Frequency
Posterior prediction intervals
Substitution rate
Relative position
Subs. type
C->T
G->A
Others

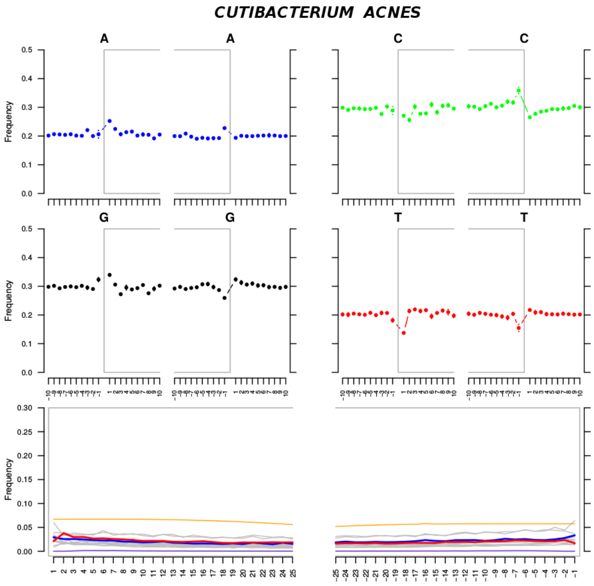
CUTIBACTERIUM ACNES
A
A
C
C
G
G
T
T
Frequency

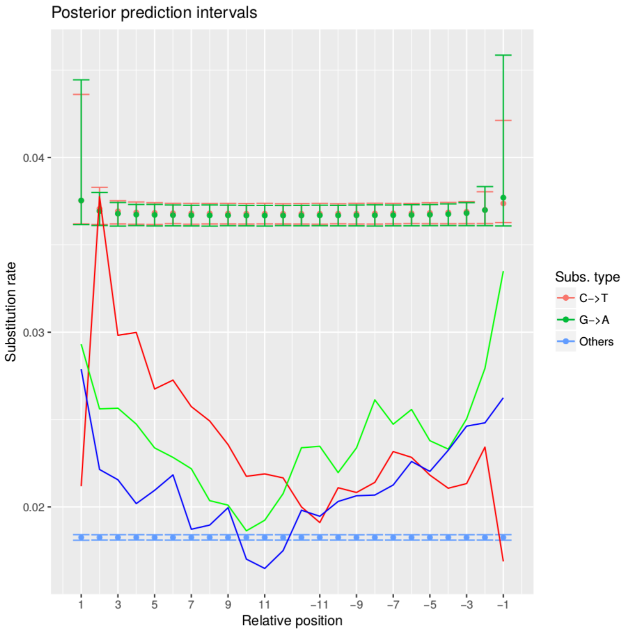
Posterior prediction intervals
Substitution rate
Relative position
Subs. type
C->T
G->A
Others

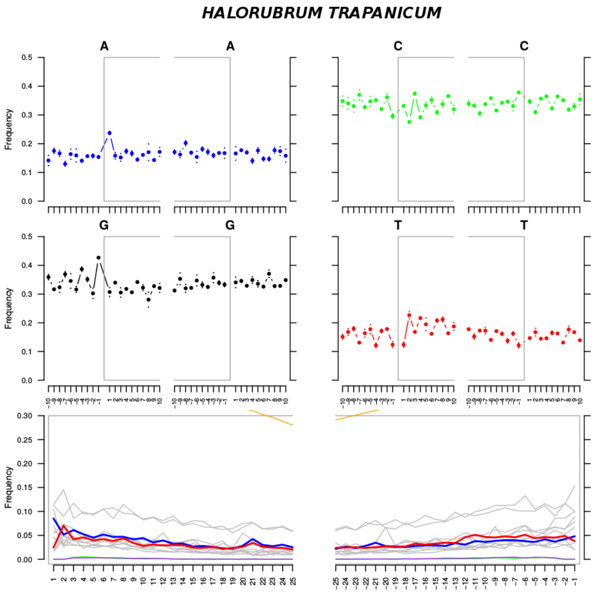
HALORUBRUM TRAPANICUM
A
A
C
C
G
G
T
T
Frequency

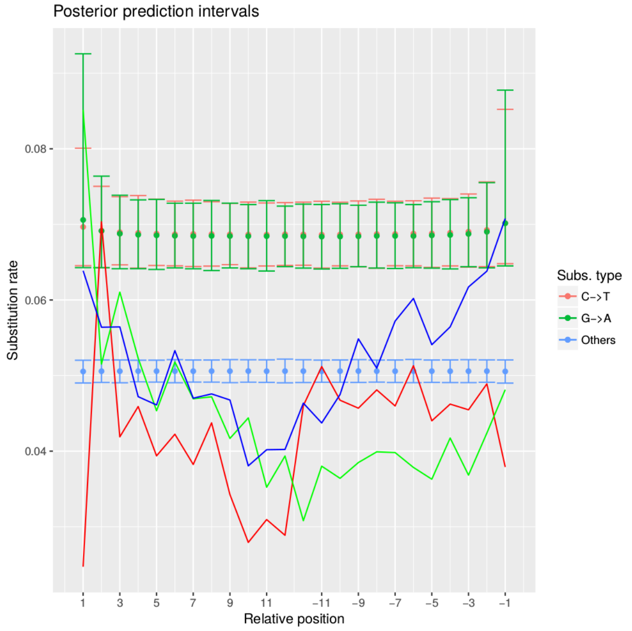
Posterior prediction intervals
Substitution rate
Relative position
Subs. type
C->T
G->A
Others

DAUCUS CAROTA

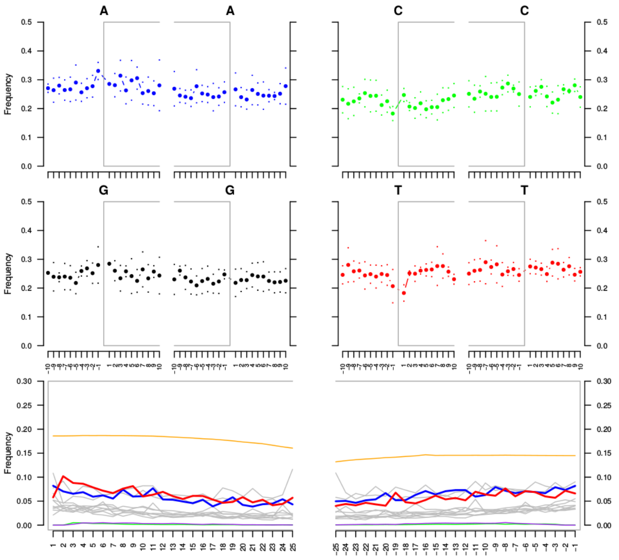

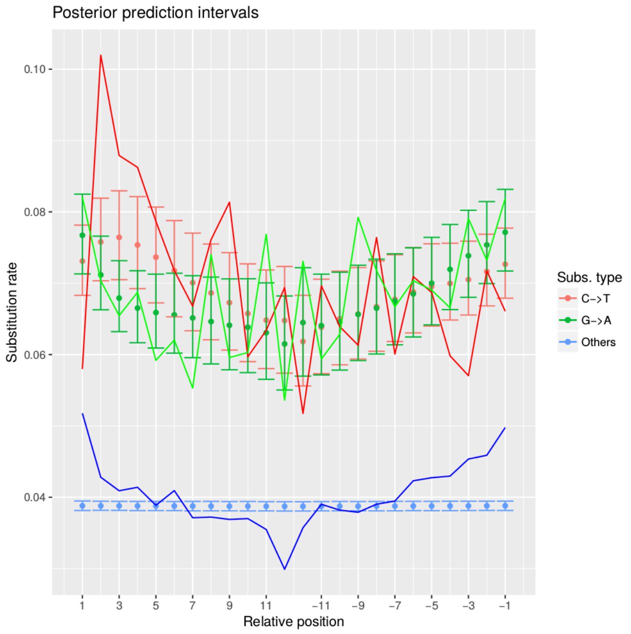

SOLANUM LYCOPERSICUM

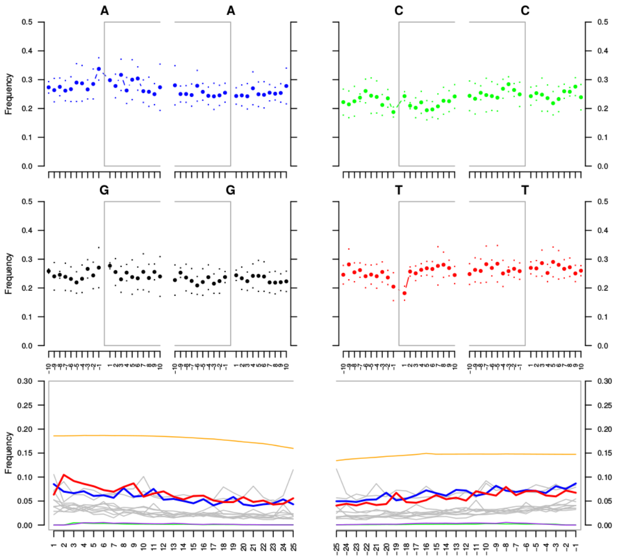

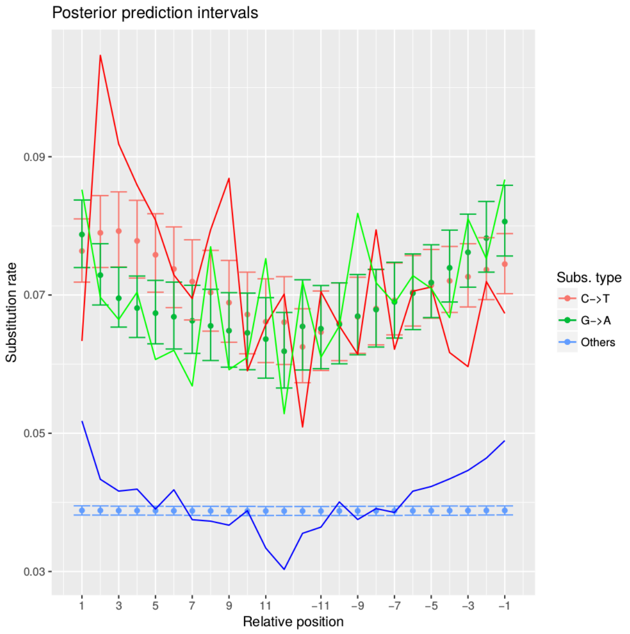

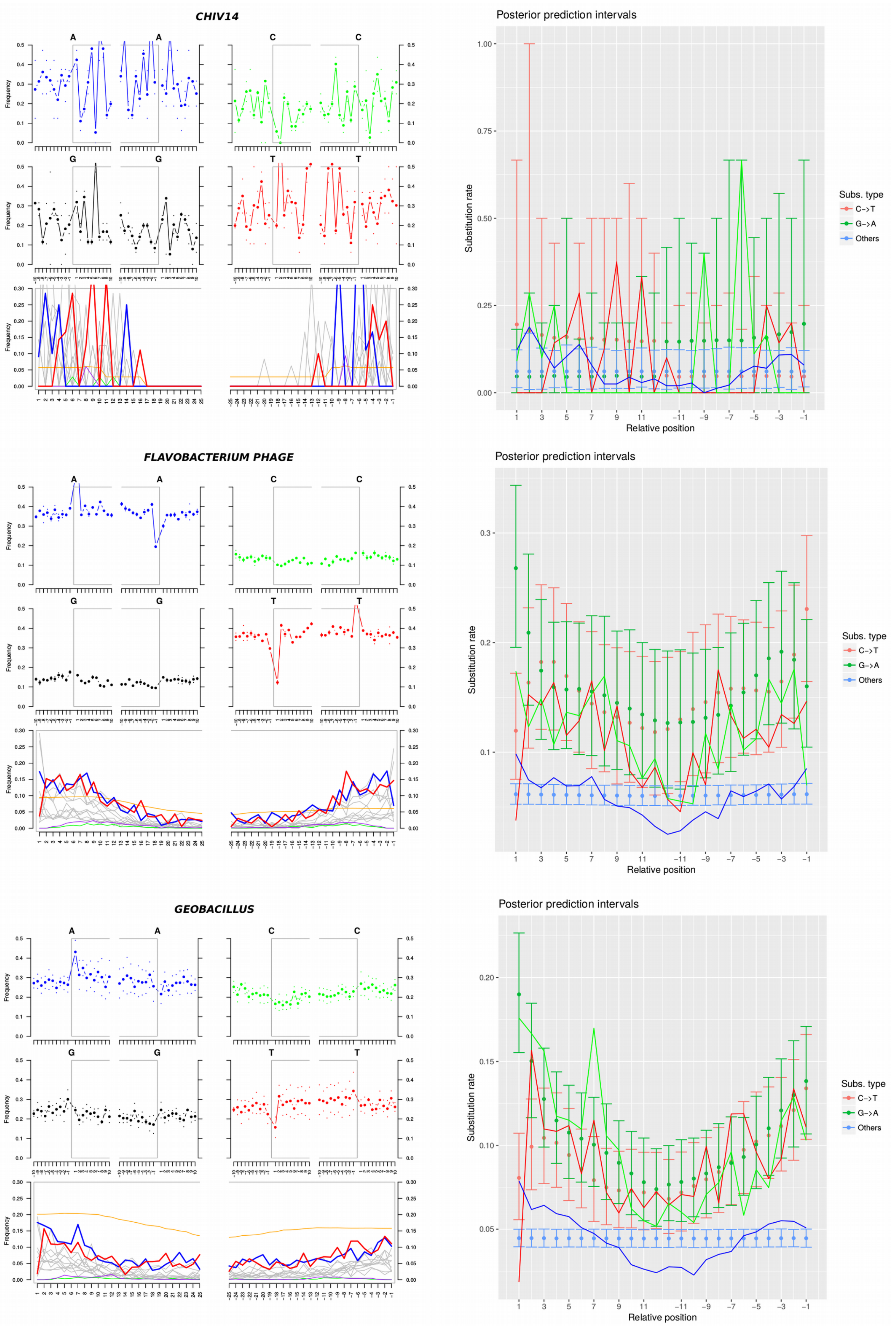

CHIV14
FLAVOBACTERIUM PHAGE
GEOBACILLUS
A
C
G
T
Frequency
Posterior prediction intervals
Substitution rate
Relative position
Subs. type
C->T
G->A
Others
